# BDE47 induces rat CYP3A1 by targeting the transcriptional regulation of miR-23b

**DOI:** 10.1038/srep31958

**Published:** 2016-08-22

**Authors:** Zhenzhen Sun, Zhan Zhang, Minghui Ji, Hongbao Yang, Meghan Cromie, Jun Gu, Chao Wang, Lu Yang, Yongquan Yu, Weimin Gao, Shou-Lin Wang

**Affiliations:** 1 Key Lab of Modern Toxicology of Ministry of Education, School of Public Health, Nanjing Medical University, 101 Longmian Avenue, Nanjing 211166, P. R. China; 2 State Key Lab of Reproductive Medicine, Institute of Toxicology, Nanjing Medical University, 140 Hanzhong Rd., Nanjing 210029, P. R. China; 3 Key Laboratory of Pediatrics, Nanjing Children’s Hospital Affiliated to Nanjing Medical University, 72 Guangzhou Road, Nanjing 210008, P. R. China; 4 Center for New Drug Safety Evaluation and Research, China Pharmaceutical University, 639 Longmian Avenue, Nanjing 211166, P. R. China; 5 Department of Environmental Toxicology, The Institute of Environmental and Human Health, Texas Tech University, 1207 Gilbert Drive, Lubbock, TX 79416, USA

## Abstract

Cytochrome P450 3A (CYP3A) is the most abundant CYP450 enzyme in the liver and is involved in the metabolism of over 50% of xenobiotics. Our previous studies revealed that 2,2′,4,4′-tetrabromodiphenyl ether (BDE47) could induce rat CYP3A1 expression, but the molecular basis remains unclear. Using *in silico* analysis, we identified a potential miR-23b recognition element (MRE23b) in the 3′-UTR region of *CYP3A1* mRNA, which was verified by the luciferase assay. The miR-23b mimic and inhibitor significantly down- and up-regulated the expression of CYP3A1, respectively. Additionally, BDE47 significantly down-regulated the expression of miR-23b in rats and in hepatic H4IIE cells. Induction or blockage of *CYP3A1* by a miR-23b inhibitor or mimic could correspondingly alter BDE47-induced expression of CYP3A1 and cytotoxicity in H4IIE cells. Furthermore, LV-anti-miR-23b significantly decreased endogenous levels of miR-23b and increased the expression and activity of CYP3A1 in rat liver. LV-anti-miR-23b also significantly increased the hydroxylated metabolites of BDE47 (3-OH-BDE47, 4-OH-BDE42, and 4′-OH-BDE49) in rat serum. In conclusion, we first found that BDE47 induced rat CYP3A1 expression by targeting the transcriptional regulation of miR-23b. This study helps provide a better understanding of CYP3A regulation and offers novel clues for the role of miRNAs in the metabolism and distribution of environmental pollutants.

It is well known that the liver is a central organ in the regulation of diverse processes, among which the metabolism, secretion, storage, and detoxification of endogenous and exogenous substances are prominent^1^. Cytochrome P450 (CYP) is a group of phase I metabolic enzymes that play a critical role in the oxidative metabolism of drugs and other xenobiotics^2^. CYP3A4 not only accounts for 30% of total human liver CYP450s, but it also is the most abundant hepatic CYP450 isoform involved in the biotransformation of various drugs and environmental chemicals. Similarly, the rat hepatic CYP3A subfamily has been widely examined in various non-clinical studies on drug metabolism, and the obtained findings are often used to estimate altered drug metabolism in humans in clinical situations^3^. CYP3A1 is the rat orthologue of CYP3A4, having 73% amino acid homology with human CYP3A4, it is also regarded as the most metabolically relevant isoforms in rats^4^.

Generally, most CYPs are transcriptionally regulated by nuclear receptors. CYP3A expression is regulated by ligand activated nuclear receptors such as pregnane X receptor (PXR), constitutive androstane receptor (CAR), and hepatocyte nuclear factor-4 alpha (HNF4α)^5^,^6^. Currently, the importance of microRNAs (miRNAs) in regulating CYPs and nuclear receptors or other transcription factors is beginning to be recognized. miR-577, miR-1, miR-532-3p, and miR-627 significantly down-regulate the translation efficiency of CYP3A4 mRNA in the liver^7^. Different levels of CYP3A4 transcription may cause substantial inter-individual variability in the metabolism of drugs and result in distinct drug effects. As a prototypical inducer, dexamethasone (DEX) could markedly increase the expression and enzymatic activity of CYP3A1 through PXR in healthy and cirrhotic rats, irrespective of the degree of liver dysfunction^8^. The dysregulation of specific miRNAs might lead to changes in drug metabolism potency or pharmacokinetics, as well as pathophysiological events^9^,^10^. Meanwhile, the circulating miRNAs could serve as potential biomarkers of liver injury in various acute and chronic liver diseases^11^.

2,2′,4,4′-tetrabromodiphenyl ether (BDE47), the dominant congener of polybrominated diphenyl ethers (PBDEs), has been identified as a developmental, reproductive, and neurological toxicant and disruptor of multiple endocrine systems in animals^12^. BDE47 was recognized as one of the substrates of CYP3A^13^. Our previous study demonstrated that BDE47 increased the expression of CYP3A1 in rat liver, and in turn affected CYP3A1-mediated metabolic activation of BDE47^14^. However, the molecular mechanism of CYP3A1-induction mediated by BDE47, especially posttranscriptional regulation, remains to be clarified.

In the present study, we first examined the metabolic activation of BDE47 by CYP3A1. Then, we found that miR-23b targets the 3′-UTR of *CYP3A1* by bioinformatic analysis and luciferase reporter assay. Subsequently, we validated the regulation of miR-23b in BDE47-induced expression and activity of CYP3A1 in *in vitro* and *in vivo* experiments. This study may provide a better understanding of CYP3A regulation and offer novel clues for the role of miRNAs in the metabolism and distribution of drugs and environmental pollutants.

## Results

### CYP3A1 induction by BDE47 in H4IIE cells

**Figure 1:**
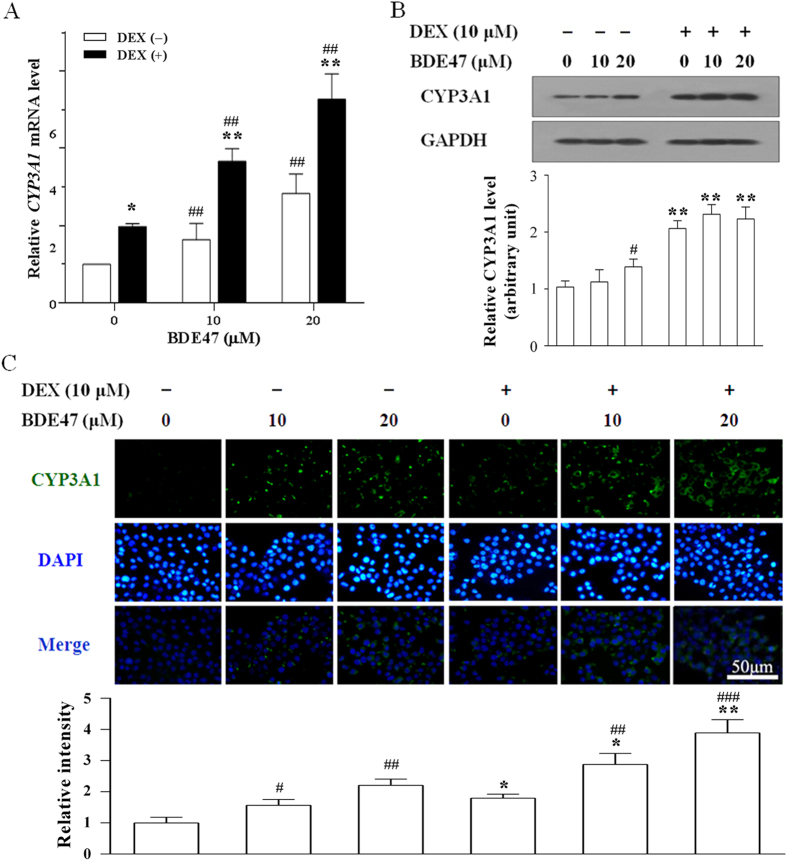
Expression and location of CYP3A1 in H4IIE cells treated with BDE47. H4IIE cells were pretreated with 10 μM DEX for 12 h, and then treated with 10 or 20 μM BDE47 for an additional 24 h. **(A)** Expression of *CYP3A1* mRNA induced by BDE47. **(B)** Expression of CYP3A1 protein induced by BDE47. **(C)** Representative fluorescent images and fluorescence intensity of CYP3A1 protein in H4IIE cells. The data is expressed as the mean ± SD of three independent experiments with triplicate samples. *P < 0.05; **P < 0.01, compared with the corresponding BDE47 treatment without DEX pretreatment; ^#^*P* < 0.05, ^##^*P* < 0.01, ^###^*P* < 0.001, compared with the corresponding vehicle control.

To avoid the cytotoxic effect of BDE47 on the evaluation of CYP3A1 expression, we first conducted a pilot experiment to determine an appropriate concentration of BDE47. As shown in Fig. S1, 20 μM BDE47 and 10 μM DEX (an inducer of CYP3A) showed no cytotoxic effects in H4IIE cells (rat hepatoma cells), and the tested concentrations were deemed suitable for the additional experiments. As expected, BDE47 dose-dependently increased *CYP3A1* mRNA (Fig. 1A) and protein expression (Fig. 1B), both of which were aggravated by DEX. The induction of CYP3A1 by BDE47 was further confirmed by immunofluorescence assay (Fig. 1C).

### Effects of CYP3A1 on BDE47 cytotoxicity in H4IIE cells

**Figure 2:**
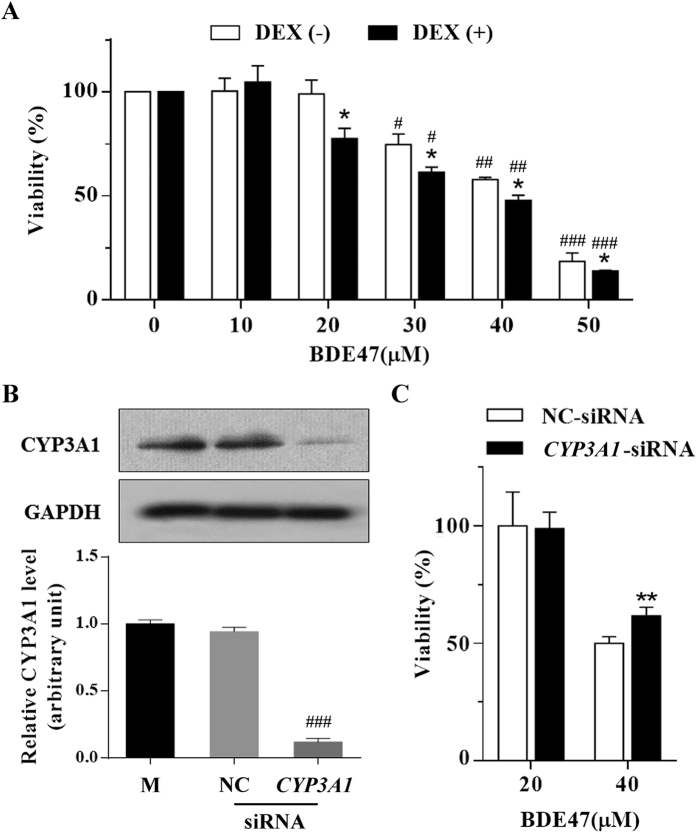
Effects of CYP3A1 on BDE47- induced cytotoxicity in H4IIE cells. **(A)** Cytotoxicity induced by BDE47. H4IIE cells were pretreated with 10 μM DEX for 12 h, and then treated with 0–50 μM BDE47 for an additional 24 h. **(B)** Knockdown of CYP3A1 expression using siRNA. H4IIE cells were transfected with 50 nM control siRNA or *CYP3A1*-siRNA for 48 h. **(C)** Effects of *CYP3A1*-siRNA on BDE47-induced cytotoxicity. H4IIE cells were transfected with 50 nM control siRNA or *CYP3A1*-siRNA for 24 h and treated with 20 or 40 μM BDE47 for an additional 24 h. DMSO (0.1%) was used as a vehicle control. Cell viability is expressed as the mean ± SD of three independent experiments with triplicate samples. *P < 0.05; **P < 0.01, compared with the corresponding BDE47 treatment without DEX pretreatment; ^#^*P* < 0.05; ^##^*P* < 0.01; ^###^*P* < 0.001, compared with the corresponding vehicle control.

As shown in Fig. 2A, BDE47 dose-dependently increased the cytotoxicity of H4IIE cells, which was enhanced by the pretreatment of DEX, an inducer of CYP3A. For example, pretreatment with DEX significantly increased 20 μM BDE47-induced cytotoxicity while no cytotoxicity was observed in the BDE47 treatment alone (Fig. 2A), indicating that CYP3A1 contributed to BDE-induced cytotoxicity. Furthermore, 40 μM BDE47-induced cytotoxicity was significantly decreased (*P* < 0.001) (Fig. 2C) after CYP3A1 expression was knocked-down by *CYP3A1*-siRNA (Fig. 2B).

### Effects of BDE47 on miR-23 expression

**Figure 3:**
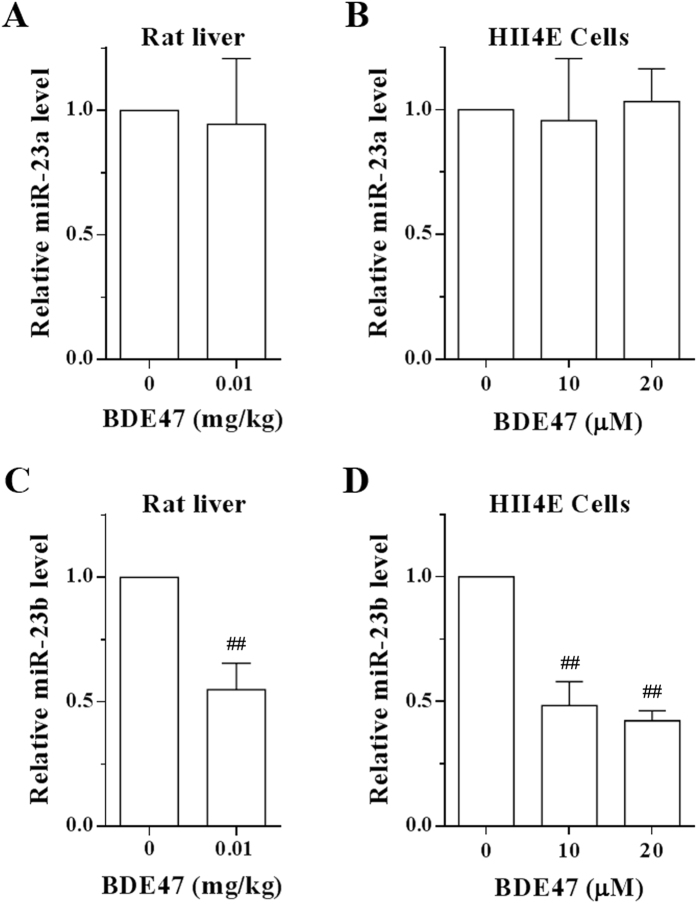
Effects of BDE47 on miR-23 expression. **(A)** Expression of miR-23a in the liver of rats treated with 0.001 mg/kg BDE47 for 8 weeks. **(B)** Expression of miR-23a in H4IIE cells treated with 10 or 20 μM BDE47 for 24 h. **(C)** Expression of miR-23b in the liver of rats treated with 0.001 mg/kg BDE47 for 8 weeks. **(D)** Expression of miR-23b in H4IIE cells treated with 10 or 20 μM BDE47 for 24 h. The data is expressed as the mean ± SD of three independent experiments with triplicate samples. ^##^*P* < 0.01, compared with vehicle control.

To investigate miRNAs in the regulation of BDE47-induced expression of CYP3A1, miRNA-target computational predictions were performed using several bioinformatic algorithms including miRanda- mirSVR^15^, miRBase. RNAhybrid^16^, miRecords, and PITA^17^. As shown in Table S1, high complementary pairing sites of miR-23 (miR-23a and miR-23b) existed in CYP3A1 3′-UTR/CDS sequences. However, miR-23a was not affected by BDE47 (Fig. 3A,B), but miR-23b significantly decreased in H4IIE cells treated with BDE47 (10 μM or 20 μM) and rat liver tissue treated with 0.001 mg/kg BDE47, generated from our previous study^14^, indicating that miR-23b instead of miR-23a is involved in the induction of CYP3A1 by BDE47 (Fig. 3C,D).

### Effects of miR-23b on CYP3A expression

**Figure 4:**
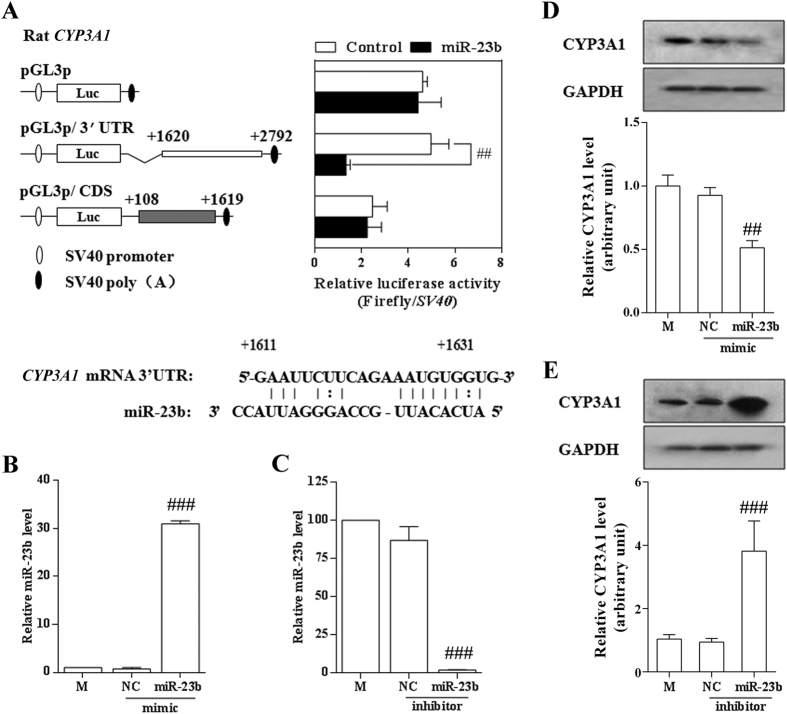
Effects of miR-23b on the expression of CYP3A1 in H4IIE cells. **(A)** Luciferase activity. Luciferase reporters with *CYP3A1* CDS or 3′-UTR were used in H4IIE cells co-transfected with miR-23b mimics or miR-control. The luciferase activity of each sample was normalized to the SV40 activity. **(B)** Effects of miR-23b mimic on the expression of miR-23b. H4IIE cells were treated with mock (M), miR-23b mimic or negative control (NC) for 24 h. **(C)** Effects of miR-23b inhibitor on the expression of miR-23b. **(D)** Effects of miR-23b mimic on the expression of CYP3A1. **(E)** Effects of miR-23b inhibitor on the expression of CYP3A1. H4IIE cells were treated with mock, miR-23b inhibitor, or negative control for 24 h. The data is expressed as the mean ± SD of three independent experiments with triplicate samples. ^##^*P* < 0.01; ^###^*P* < 0.001, compared with the corresponding control.

To investigate whether miR-23b is functional in the regulation of *CYP3A* expression, luciferase assays were performed using H4IIE cells and HepG2 cells. For rat *CYP3A1*, the reporter activities of pGL3p/*CYP3A1* 3′-UTR (+1620–+2792) instead of pGL3p/CDS (+108–+1619) decreased significantly in comparison to the control plasmid (Fig. 4A). For human *CYP3A4*, as predicted for the complementary pairing sites of miR-23b and *CYP3A4* (Fig. S2A), the reporter activities of the pGL3p/*CYP3A4* CDS (+80–+1588), particularly CDS (+450–+750), (+1150–+1400), and (+1490–+1710) (Fig. S2C), were significantly lower than that of the control plasmid (Fig. S2B, C), which was further confirmed by the results for the mutants of their corresponding CDS regions (Fig. S2D). These data suggest that miR-23b potentially regulates the expression of rat *CYP3A1* and human *CYP3A4*. To validate these findings, the pGL3p/3′UTR plasmid was co-transfected with miR-23b mimic or a miR-23b inhibitor into H4IIE cells. The miR-23b mimic increased the intracellular miR-23b level (Fig. 4B), and down-regulated the expression of CYP3A1(Fig. 4D). Correspondingly, the miR-23b inhibitor decreased the intracellular miR-23b level (Fig. 4C), and up-regulated CYP3A1 expression (Fig. 4E).

### Regulation of miR-23b on BDE47-induced CYP3A1 expression and cytotoxicity in H4IIE cells

**Figure 5:**
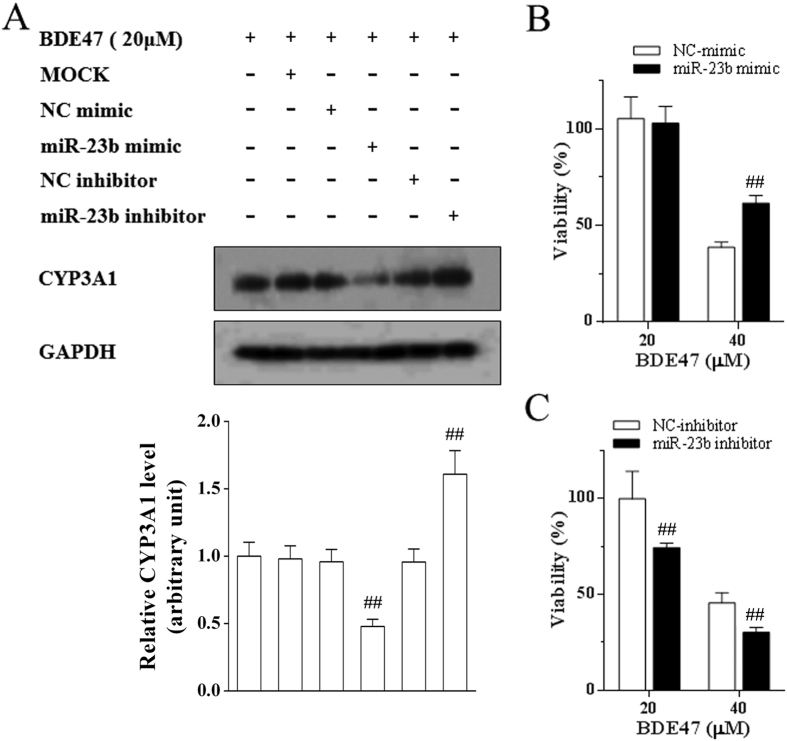
Effects of miR-23b on BDE47-induced expression of CYP3A1 and cytotoxicity in H4IIE cells. **(A)** Expression of CYP3A1. H4IIE cells were treated with 20 μM BDE47 for 24 h after miR-23b mimic or miR-23b inhibitor pretreatment for 24 h. **(B)** Effects of miR-23b mimic on BDE47-induced cytotoxicity. H4IIE cells were treated with 20 or 40 μM BDE47 for 24 h after miR-23b mimic pretreatment for 24 h. **(C)** Effects of miR-23b inhibitor on BDE47-induced cytotoxicity. H4IIE cells were treated with 20 or 40 μM BDE47 for 24 h after miR-23b inhibitor pretreatment for 24 h. The data is expressed as the mean ± SD of three independent experiments with triplicate samples. ^##^*P* < 0.01, compared with the negative control for miR-23b mimics or inhibitor.

Pretreatment of miR-23b mimic significantly inhibited CYP3A1 expression in H4IIE cells treated with BDE47, while the miR-23b inhibitor enhanced expression (Fig. 5A). Moreover, the miR-23b mimic reversed and the miR-23b inhibitor aggravated the decrease in the viability of H4IIE cells treated with BDE47 (Fig. 5B,C), providing evidence that BDE47-mediated miR-23b induced CYP3A1, and subsequent cytotoxicity of H4IIE cells.

### Effects of miR-23b on the expression and activity of CYP3A1 in rats treated with BDE47

**Figure 6:**
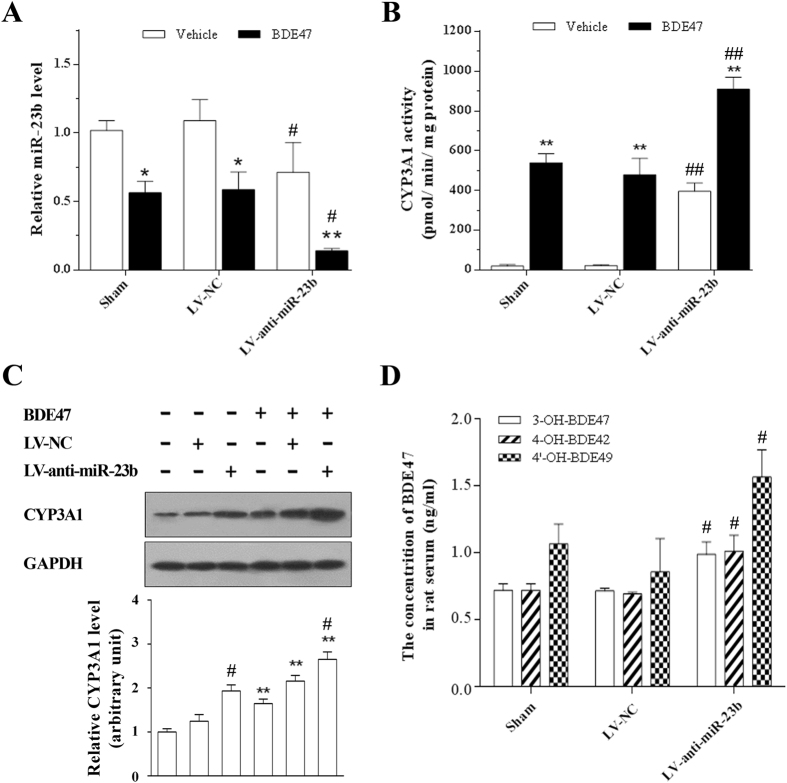
Effects of miR-23b on BDE47-induced CYP3A1 expression and activity in rats. Thirty rats were randomly divided into three groups and were given an intravenous injection of 500 μL PBS solution, Lentiviral-negative control (LV-NC), or LV-anti-miR-23b. After 3 days, each group was randomly divided into two subgroups and were given corn oil or BDE47 (5 mg/kg) by gavage for 7 continuous days. **(A)** Expression of miR-23b in rat liver. **(B)** Effects of miR-23b on hepatic CYP3A-related 7-ethoxyresorufin O-deethylase (EROD) activity. **(C)** Effects of miR-23b on CYP3A1 expression in rat liver. Hepatic microsome protein (10 μg) from each sample was used in the immunoblotting assay. GAPDH was used as an internal reference. **(D)** Effects of miR-23b on the hydroxylated metabolites of BDE47 in rat serum. The data is expressed as the mean ± SD of ten samples. *P < 0.05; **P < 0.01, compared with the corresponding vehicle control; ^#^*P* < 0.05; ^##^*P* < 0.01, compared with Sham and LV-NC controls.

To further determine the role of miR-23b in the function of CYP3A1 in the liver, rats received a caudal vein injection of LV-anti-miR-23b or LV-NC, which were chosen for their effects on miR-23b and CYP3A1 expression in H4IIE cells (Fig. S3). As expected, LV-anti-miR-23b alone not only significantly decreased miR-23b levels, but also increased the expression and activity of CYP3A1 in rat liver. BDE47 treatment alone showed similar results, and those findings were further augmented in the co-treatment of BDE47 and LV-anti-miR-23b in rats, which exemplified the important role of miR-23b in BDE47-induced expression and activity of CYP3A1 (Fig. 6A–C).

CYP3A is regarded as one of the major metabolic enzymes of BDE47, thus BDE47 and its hydroxylated metabolites in rat serum were measured to further evaluate the role of miR-23b in the function of CYP3A1. As shown in Fig. 6D, the hydroxylated metabolites of BDE47, including 3-OH-BDE47, 4′-OH-BDE49, and 4-OH-BDE42, were found in rat serum, and all of them significantly increased in the LV-anti-miR-23b group compared to the control group (Sham or LV-NC) (*P* < 0.05). These results suggest that miR-23b contributed to the metabolism of BDE47 through the regulation of CYP3A1.

## Discussion

Based on *in vitro* and *in vivo* experiments, we first investigated the role of miR-23b in the BDE47-induced expression and activity of CYP3A1. Bioinformatics analysis identified a potential miR-23b recognition element (MRE23b) in rat *CYP3A1* and human *CYP3A4* mRNA. The luciferase assay revealed that endogenous and exogenous miR-23b negatively regulated the activity through MRE23b. miR-23b not only regulated BDE47-induced expression of CYP3A1 and corresponding cytotoxicity of H4IIE cells, but it also regulated the expression and activity of CYP3A1, as well as the oxidative metabolism of BDE47 in rats. These results clearly illustrate that miR-23b acts as a miRNA that targets *CYP3A1* mRNA and it plays an important role in the BDE47-induced expression and activity of CYP3A by targeting transcriptional regulation.

Typically, the most common mechanism of CYP induction is transcriptional gene activation. Previous studies have shown that drugs and environmental chemicals indirectly regulate the expression and activity of CYP enzymes by regulating the nuclear receptor family and other transcription factors. The nuclear receptor family is involved in the regulation of CYP enzymes induced by xenobiotics, and PXR and CAR have been confirmed as classical pathways^18^,^19^. Exposure to BDE47 induced the expression of *CYP1A1*, *CYP2B*, and *CYP3A* in F344 rats^20^ and our previous study revealed that DEX could enhance the BDE47-induced expression and activity of CYP3A in the liver of rats^14^. BDE47 could induce CYP3A4 genes through the activation of CAR and PXR in human primary hepatocytes^21^. It has been verified that H4IIE cells are suitable for evaluating the potential of a drug or compound to induce CYP3A23 expression^22^. In the present study, we reconfirmed that BDE47 significantly induced the expression and activity of CYP3A1 in both H4IIE cells and rat liver.

In recent years, it was discovered that miRNAs might have a direct or indirect effect on xenobiotic regulation of CYP enzymes^9^,^23^. miR-148a could cause post-transcriptional repression of PXR expression by targeting its 3′-UTR, which further impacts *CYP3A4* induction^24^. miR-27b and miR-298 not only inhibit post-transcriptional *CYP3A4* expression, but they also indirectly regulate the expression of CYP3A4 through the inhibition of the vitamin D receptor (VDR)^25^. However, the role of miRNAs in the BDE47-induced expression of CYP3A remains unclear.

There is growing evidence indicating that miRNAs play an important role in toxicogenomics, disease etiology, and the effect of toxicants^26^. miR-23b has been reported as a small RNA with a broad regulatory role, for example, it is known to regulate gene expression and anti-oxidant or pro-oxidant pathways, and it plays a role in tumor development^27^. miR-23b inhibited the generation of reactive oxygen species by inhibiting the expression of NOX4, a member of the NADPH oxidase family^28^, it was also found to trigger cancer-promoting effects by inhibiting the expression of apoptosis antigen 1 (FAS) and the tumor suppression gene, phosphatase and tensin homolog (PTEN), in lymphoma and kidney cancer^29^,^30^. However, the effect of miR-23b on the expression of CYP enzymes is still unknown. Using bioinformatics software, miR-23a and miR-23b could target the nucleic acid sequence of rat *CYP3A1* but only miR-23b was affected by BDE47, and miR-23b could directly bind to the 3′-UTR region of *CYP3A1* and inhibit the expression of CYP3A1. These results suggest that miR-23b functionally recognizes the 3′-UTR region on the rat *CYP3A1* mRNA. Interestingly, rat *CYP3A1* is homologous to human *CYP3A4*, and they both share functions involved with chemical metabolism. However, the recognition sites of miR-23b exist in the CDS regions of CYP3A4 mRNA, the mechanism and effects need to be further elucidated.

Recently, studies have focused on miRNA-dependent regulation of drug-metabolizing enzymes and nuclear receptors, and the associated potential toxicological implications^31^. In the present study, miR-23b was found to inhibit the metabolic activation of BDE47 in H4IIE cells, which was further confirmed in rats treated with BDE47. Blockage of miR-23b by Lv-anti-miR-23b significantly increased the expression and activity of CYP3A1 in liver microsomes of rats treated with BDE47, and it also increased oxidative metabolites of BDE47 (3-OH-BDE47, 4-OH-BDE47, and 4′-OH-BDE47) in the serum, further revealing the important role of miR-23b in BDE47-induced expression and activity of CYP3A1.

This study provides new insight into the unsolved mechanism of the post-transcriptional regulation of CYP450 enzymes. The utilization of miRNAs may open a new era in the fields of metabolism, pharmacokinetics/toxicokinetics, and toxicology for drugs and environmental pollutants. Furthermore, intervention in miRNA pathways can alter the sensitivity of cells to xenobiotics, which could further expound drug-drug interactions and help to avoid drug-related side effects and provide a new strategy for the prevention and control of environmental pollutants.

## Methods

### Cell viability assay and transfection

H4IIE cells were obtained from the Chinese Academy of Medical Sciences, Institute of Basic Medical Cell Center (Beijing, China). H4IIE cells were plated into 96-well plates (5000 cells/well) with 200 μL of Minimum Essential Medium with Earle’s Balanced Salt Solution (MEM-EBSS) (GIBCO-BRL, Grand Island, NY, USA) and cultured at 37 °C in 5% CO_2_ overnight. Then the cells were treated with 0–100 μM BDE47 (purity of ≥98.7%, Chemservice, West Chester, PA, USA) for 24 h. DEX, an inducer of CYP3A1, was obtained from Sigma-Aldrich, St. Louis, MO, USA. To investigate the role of CYP3A1 in BDE47-induced cytotoxicity, H4IIE cells were pretreated with 10 μM DEX for 12 h, and then treated with 0–50 μM BDE47 for an additional 24 h. Control short interfering RNA (siRNA) and *CYP3A1* siRNA were purchased from GENERAY Biotechnology (Shanghai, China). miR-23b mimic, miR-23b inhibitor, and miRNA mimic and inhibitor negative control (NC) were purchased from RiBoBio (Guangzhou, China). The cells were transfected using Lipofectamine 2000 (Invitrogen, Carlsbad, CA) according to the manufacturer’s protocol, and cells were harvested 24 h after transfection. After the treatment, cell viability was determined using the CCK-8 assay as described previously^32^.

### Immunofluorescence assay

H4IIE cells were seeded in 6-well plates (4 × 10^5 ^cells/well). Cells were pretreated with 10 μM DEX for 12 h, and then treated with 0, 10, or 20 μM BDE47 for an additional 24 h. After treatment, the cells were incubated with CYP3A1 antibody at 4 °C overnight and then incubated with fluorscein isothiocyanate (FITC)-conjugated secondary antibody at room temperature for 1 h. 4′,6-diamidino-2-phenylindole (DAPI) was added to stain the nuclei for 30 seconds. The immunofluorescence of cells was examined using a fluorescence microscope (Olympus, Tokyo, Japan).

### Luciferase reporter assay

Luciferase reporter plasmids were constructed by GENERAY Biotechnology (Shanghai, China). Various fragments containing the miR-23b MRE were inserted at the *Xba* I site downstream of the luciferase gene in the pGL3-promoter vector. The fragments used in the experiments were *CYP3A1* coding sequence (CDS, from +108 to +1619) and *CYP3A1* 3′-UTR (from +1620 to +2792). For the luciferase reporter assay, 5 × 10^3^ H4IIE cells or HepG2 cells were seeded in 24-well plates and then were co-transfected with the luciferase reporter plasmids (0.8 μg/well), SV40, and miR-23b mimic or mimic NC (100 nM) using Lipofectamine 2000 according to the manufacturer’s protocol. Luciferase activity was measured at 24 h after transfection using the dual-luciferase reporter assay system (Promega, Madison, WI) with a luminometer (Lumat LB960, Berthold Tech., Bad Wildbad, Germany).

### Animal treatment and sample collection

Male Sprague-Dawley rats (4–5 weeks) were obtained from Shanghai SLAC Laboratory Animal Co., Ltd (Shanghai, China), and maintained under standard conditions (23 ± 2 °C) with 12 h light/dark cycles. The animals were given a standard pellet diet and water *ad libitum* and were acclimatized for at least 1 week prior to use. Thirty rats were randomly divided into three groups and were given an intravenous injection of 500 μL PBS solution, Lentiviral-negative control (LV-NC, 5 × 10^7 ^TU), or Lentiviral-anti-miR-23b (LV-anti-miR-23b, 5 × 10^7 ^TU). Each group was randomly divided into two subgroups, which were given corn oil (control) and BDE47 (5 mg/kg) by gavage continuously for seven days. After the treatment, the rats were euthanized by CO_2_ asphyxiation after fasting for 12 h. Cardiac blood and liver were immediately separated and stored at −80 °C until further needed. All procedures were approved by the Animal Care and Use Committee of Nanjing Medical University, and were performed in accordance with the Animal Management Rules of the National Health and Family Planning Commission of the People’s Republic of China.

### Detection of CYP3A1 activity in rat liver

Frozen liver samples were used to prepare the hepatic microsomes. Hepatic CYP3A-related 7-ethoxyresorufin O-deethylase (EROD) activity was measured using a fluorescence assay kit (Genmed Scientifics, Shanghai, China) according to our previous study 14. Briefly, 80 mg/mL of the microsomal protein was used in the metabolic reactions and 7-benzyloxyresorufin was used as the probe substrate for 7-benzyloxyresorufin O-dealkylatase (CYP3A1). The fluorescence intensity (λ_ex_ = 530 nm, λ_em_ = 590 nm) was read by an Infinite M200 plate reader (Tecan, Seestrasse, Männedorf, Switzerland).

### Detection of BDE47 hydroxylated metabolites in rat serum

The BDE47 hydroxylated metabolites in rat serum were detected according to our previous study^14^. Briefly, the hydroxylated metabolites of BDE47 were extracted by n-hexane and methyl tert-butyl ether (1:1), and all of the samples were washed using a florisil solid phase extraction (SPE) cartridge eluted with dichloromethane: hexane (1:1). Toluene, pyridine, and acetic anhydride were used for the derivatization of these hydroxylated metabolites. The sample was vortexed and back-extracted with hexane, nitrogen-evaporated to near dryness, and then reconstituted in dichloromethane. The analysis of the BDE47 hydroxylated metabolites was performed on a gas chromatograph-mass spectrometer (Thermo Finnigan DSQ, USA).

### Quantitative real-time PCR (q-PCR)

Total RNA was extracted from H4IIE cells and rat livers, then cDNA was synthesized by the Transcriptor First Strand cDNA Synthesis Kit (Roche, Mannheim, Germany), and q-PCR was performed using the FastStart Universal SYBR Green Master Kit (Roche, Mannheim, Germany). All the miRNA cDNA was synthesized with the One Step PrimeScript^®^ miRNA cDNA Synthesis Kit (Takara, Kyoto, Japan) and then SYBR^®^ Premix Ex Taq^TM^ II (Takara, Kyoto, Japan) was used for q-PCR with the forward primer only. The Uni-miR qPCR primer that is contained in the One Step PrimeScript^®^ miRNA cDNA Synthesis Kit was used for the reverse primer. *GAPDH* and *U6* were used as the reference genes to normalize expression levels of common mRNA or miRNA, respectively. Both mRNA and the miRNA-specific primers were designed and synthesized by GENERAY Biotechnology (Shanghai, China), and the primer sequences are listed in Table S2. q-PCR was performed using an ABI 7300 Fast Real Time PCR system (Applied Biosystems, Foster City, CA, USA). Relative gene expression was analyzed according to the 2^−ΔΔCt^ method as described previously^14^.

### Western blots

The expression of CYP3A1 in liver microsomes was determined according to our previous study^14^. Equal amounts of protein (20 μg microsomes or 80 μg cell lysates) were separated by sodium dodecyl sulfate polyacrylamide gel electrophoresis (SDS-PAGE) on 12.5% polyacrylamide gels and were transferred to polyvinylidene difluoride membranes (Millipore, Billerica, MA, USA). Using a specific antibody for CYP3A1 (Millipore, Billerica, MA, USA), the protein immune complexes were detected by an enhanced chemiluminescence (ECL) immunoblotting assay kit and exposed to Kodak X-Omat film. The signals obtained from the western blot analysis were quantified with Image J software (http://rsb.info.nih.gov/ij), as described previously^32^.

### Statistical analysis

Statistical analysis was conducted using SPSS version 21.0 (Chicago, IL, USA). All data are represented as the mean ± SD of at least three independent experiments. One-way analysis of variance (ANOVA) was performed to assess the differences among groups. *P* < 0.05 was considered statistically significant.

## Additional Information

**How to cite this article**: Sun, Z. *et al.* BDE47 induces rat CYP3A1 by targeting the transcriptional regulation of miR-23b. *Sci. Rep.* **6**, 31958; doi: 10.1038/srep31958 (2016).

## Supplementary Material

Supplementary Information
