# Supplementary material for: BDE47 induces rat CYP3A1 by targeting the transcriptional regulation of miR-23b
Source: Sci Rep. 2016 Aug 22;6:31958. doi: 10.1038/srep31958 (PMC4992956; doi:10.1038/srep31958)
Supplement: Supplementary Information [file srep31958-s1.pdf]

BDE47 induces rat CYP3A1 by targeting the transcriptional regulation of miR-23b

Zhenzhen Sun, Zhan Zhang, Minghui Ji, Hongbao Yang, Meghan Cromie, Jun Gu, Chao Wang, Lu Yang, Yongquan Yu, Weimin Gao, Shou-Lin Wang

Supplementary Information

Table S1. miRNA-target computational predictions using miRanda-mirSVR

Target mRNA	Conserved miRNAs	mirSVR score
CYP3A1	rno-miR -23a	-1.2356
CYP3A1	rno-miR-23b	-1.2356
CYP3A1	rno-miR-382	-0.8601
CYP3A1	rno-miR-7a	-0.458
CYP3A1	rno-miR-7c	-0.458
CYP3A1	rno-miR-7d	-0.458
CYP3A1	rno-let-7e	-0.458
CYP3A1	rno-let-7f	-0.458
CYP3A1	rno-miR-98	-0.458
CYP3A1	rno-let-7b	-0.4547
CYP3A1	rno-let-7i	-0.4547
CYP3A1	rno-miR-186	-0.3108
CYP3A1	rno-miR-443	-0.2215
CYP3A1	rno-miR-194	-0.1742
CYP3A1	rno-miR-196b	-0.1542
CYP3A1	rno-miR-196c	-0.1542
CYP3A1	rno-miR-196a	-0.1527
CYP3A1	rno-miR-425	-0.1084

Table S2. Primer sequences used in the qRT-PCR

Gene		Sequence (5' to 3')
CYP3A1	F	ACCCACCAGCAGCACACTTT
	R	TCTCCTCCTGCAGTTTCTTCTGT
GAPDH	F	GCCGAGGGCCCACTAAAG
	R	AGCATCAAAGGTGGAAGAATGG
miR-23b	F	ATCACATTGCCAGGGATTACC
U6	F	CTCGCTTCGGCAGCACA
siRNA- CYP3A1	F	GCACUGUGCUGAAUUACUA dTdT
	R	dTdT CGUGACACGACUUAAUGAU

F: forward primer; R: reverse primer.

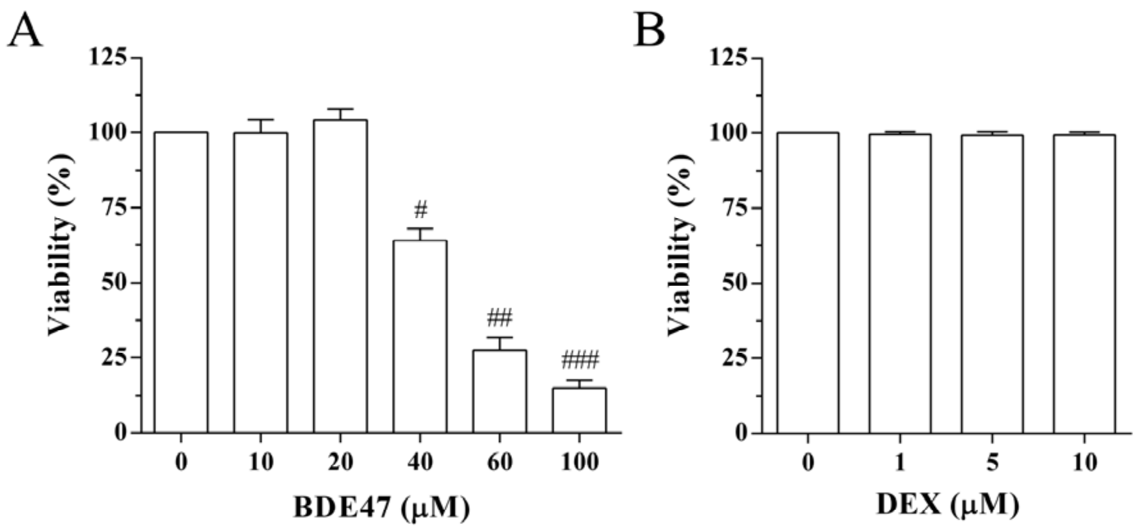

Figure S1. Initial screening of treatment concentrations of BDE47 or DEX in H4IIE cells. (A) Cell viability in BDE47 treatment for 24 h. **(B)** Cell viability in DEX treatment for 24 h. $^{\#}P < 0.05$; $^{\#\#}P < 0.01$; $^{\#\#\#}P < 0.001$, compared with vehicle control.

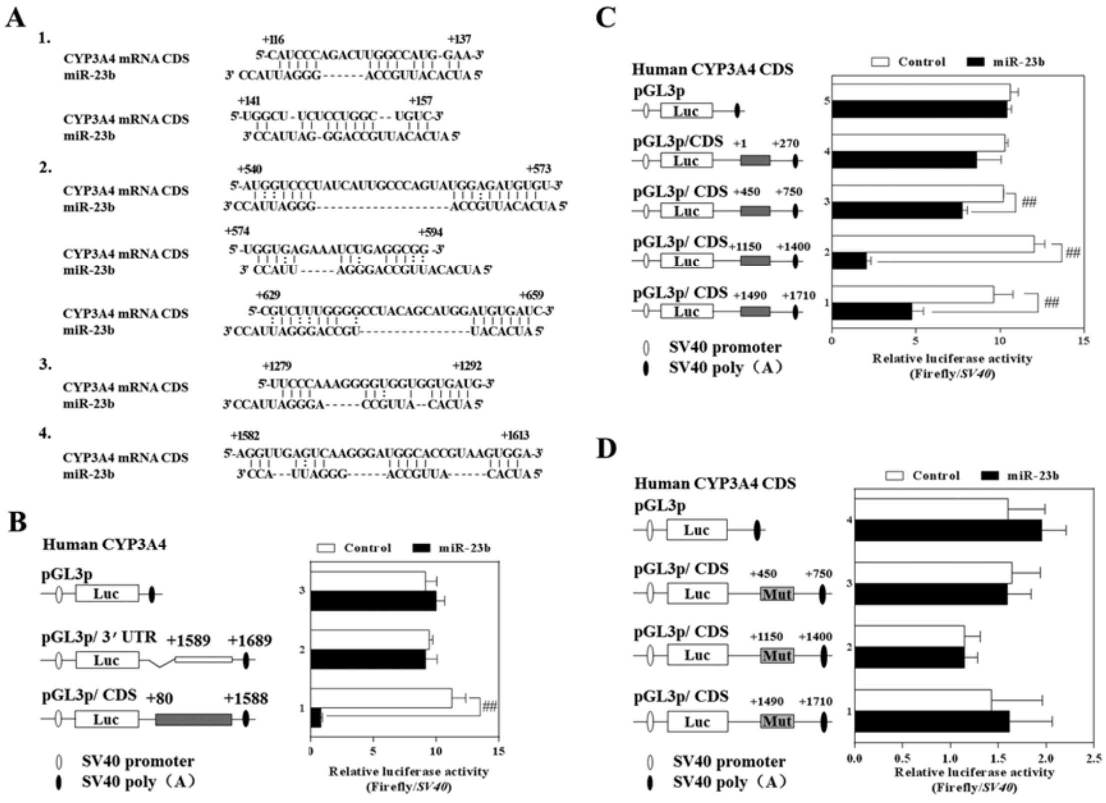

Figure S2. Luciferase activity of plasmids containing the potential recognition element of miR-23b in the 3'-UTR or CDS of human *CYP3A4* mRNA. The reporter plasmids containing the 3'-UTR or CDS of *CYP3A*4 were transiently transfected into HepG2 cells with miR-23b mimics or control. **(A)** Predicted target sequence of miR-23b in human *CYP3A4* mRNA. **(B)** Luciferase activity of pGL3p/3'-UTR (+1589- +1689) and pGL3p/CDS (+80- +1588). **(C)** Luciferase activity of pGL3p containing the different length of *CYP3A4* CDS (from +80 to +1588, from +1 to +270, from +450 to +750, from +1150 to +1400 or from +1490 to +1710). **(D)** Luciferase activity of pGL3p containing the inverted *CYP3A4* CDS (mutant) for different lengths. The luciferase activity of each sample was normalized to SV40 activity. The data is expressed as the mean ± SD of three independent experiments with triplicate samples. ##*P*< 0.01, compared with the corresponding control.

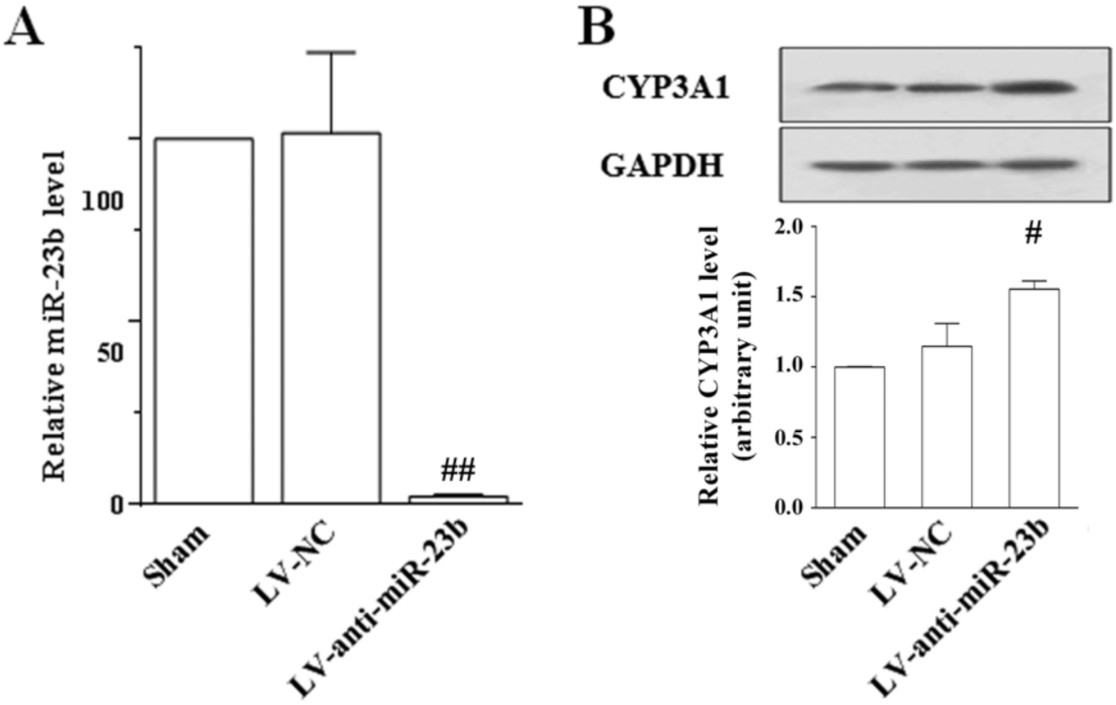

Figure S3. Initial efficiency of LV-anti-miR-23b knockdown of CYP3A1 in H4IIE cells. H4IIE cells were treated with LV-anti-miR-23b or its negative control for 24 h. **(A)** Expression of miR-23b. **(B)** Expression of CYP3A1. $^{\#}P< 0.05$, $^{\#\#}P< 0.01$, compared with Sham and LV-NC the controls.
